# Unassisted single-channel transcolonic endoscopic appendectomy for an appendiceal neuroma

**DOI:** 10.1055/a-2418-0958

**Published:** 2024-10-02

**Authors:** Pei-Rong Xu, Zu-Qiang Liu, Minying Deng, Quan-Lin Li, Pinghong Zhou

**Affiliations:** 1 92323 Endoscopy Center and Endoscopy Research Institute, Zhongshan Hospital Fudan University, Shanghai, China; 2 92323 Pathology, Zhongshan Hospital Fudan University, Shanghai, China

A 34-year-old man was admitted with recurrent abdominal pain over the preceding 2 years. A computed tomography (CT) scan revealed chronic appendicitis and an appendiceal fecal stone ( Fig. 1 ). After a comprehensive preoperative assessment had been completed, transcolonic endoscopic appendectomy was chosen.

**Fig. 1:**
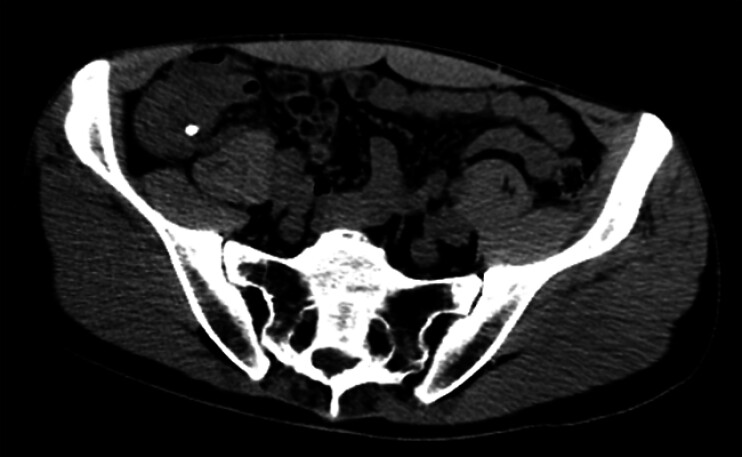
Computed tomography scan of the pelvis showing an appendiceal fecal stone with evidence of chronic appendicitis.

First, saline with indigo rouge was injected into the submucosa at the opening of the annular appendix ( Fig. 2 **a** ). A circumferential incision was then made around the appendiceal orifice as deep as the muscularis propria with a HookKnife ( Fig. 2 **b** ). Incision into the serosal layer was performed with an IT knife to create active perforation and the endoscope was then advanced into the peritoneal cavity ( Fig. 2 **c** ). The appendix was gradually separated along the meso-appendix and was resected with a snare ( Fig. 2 **d** ). After hemostasis had been carefully achieved, the post-resection defect was closed with a purse-string suture ( Fig. 2 **e** ). A drainage tube was placed at the wound area, transanally under direct vision, and fixed to the cecal mucosa. Another drainage tube was inserted transanally into the rectum for decompression. The total procedure duration was 130 minutes. The patient recovered uneventfully and was discharged on postoperative day 4. The entire procedure is shown in Video 1 .

**Fig. 2:**
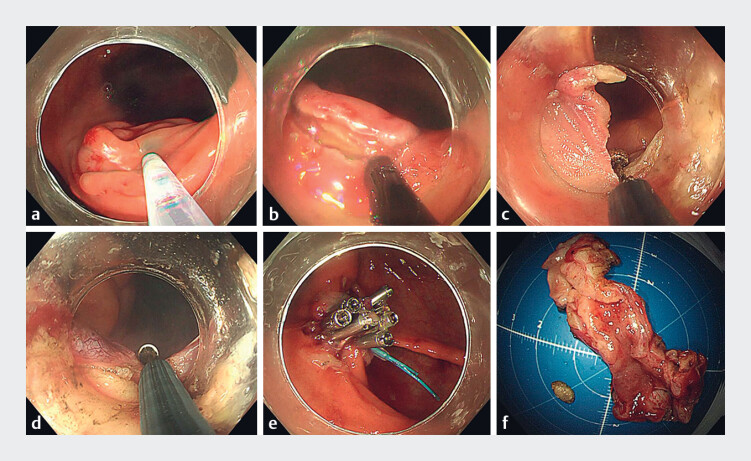
Images from the unassisted single-channel transcolonic endoscopic appendectomy of an appendiceal neuroma showing: **a** an indigo rouge and saline mixture being injected into the submucosa at the opening of the annular appendix; **b** a circumferential incision made with a HookKnife to the depth of the muscularis propria around the appendiceal orifice; **c** incision of the serosal layer made using an IT knife to create active perforation; **d** the appendix being gradually separated along the meso-appendix; **e** the defect completely closed by a purse-string suture; **f** the resected specimen.

Unassisted single-channel transcolonic endoscopic appendectomy is performed for an appendiceal neuroma.Video 1

The final pathologic diagnosis was an appendiceal neuroma. The hematoxylin and eosin (H&E)-stained sections showed a proliferation of spindle cells in the submucosa ( Fig. 3 **a** ). Most of these cells were positive for S100 protein ( Fig. 3 **b** ). Appendiceal neuroma is a rare benign lesion characterized by proliferation of neural tissue due to frequent inflammatory attacks 1 2 . The traditional treatment is open or laparoscopic appendectomy. Transcolonic endoscopic appendectomy is a new endoscopic resection technique that has developed quickly in recent years. This is the first reported case of transcolonic endoscopic appendectomy for an appendiceal neuroma; the procedure was challenging, probably owing to fibrosis of the appendix.

**Fig. 3:**
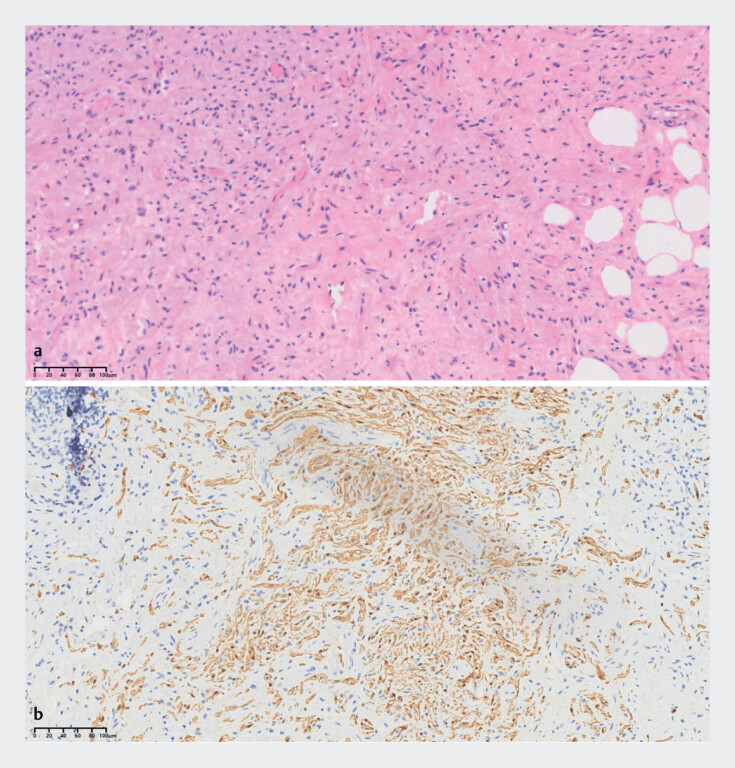
Microscopic images of the resected appendix showing: **a** on hematoxylin and eosin staining, a proliferation of spindle cells; **b** positivity for S-100.

Endoscopy_UCTN_Code_TTT_1AQ_2AD_3AF

**Correction**

**Correction: Unassisted single-channel transcolonic endoscopic appendectomy for an appendiceal neuroma** Pei-Rong Xu, Zu-Qiang Liu, Minying Deng et al. Unassisted single-channel transcolonic endoscopic appendectomy for an appendiceal neuroma. Endoscopy 2024; 56: E843–E844, doi:10.1055/a-2418-0958 In the above-mentioned article the arrangement of Figure 2 has been corrected. This was corrected in the online version on October 9, 2024.

## Notes

Endoscopy E-Videos https://eref.thieme.de/e-videos
*E-Videos* is an open access online section of the journal *Endoscopy* , reporting on interesting cases and new techniques in gastroenterological endoscopy. All papers include a high-quality video and are published with a Creative Commons CC-BY license. Endoscopy E-Videos qualify for HINARI discounts and waivers and eligibility is automatically checked during the submission process. We grant 100% waivers to articles whose corresponding authors are based in Group A countries and 50% waivers to those who are based in Group B countries as classified by Research4Life (see: https://www.research4life.org/access/eligibility/ ). This section has its own submission website at https://mc.manuscriptcentral.com/e-videos .

## Notes

Correction **Correction: Unassisted single-channel transcolonic endoscopic appendectomy for an appendiceal neuroma** Pei-Rong Xu, Zu-Qiang Liu, Minying Deng et al. Unassisted single-channel transcolonic endoscopic appendectomy for an appendiceal neuroma. Endoscopy 2024; 56: E843–E844, doi:10.1055/a-2418-0958 In the above-mentioned article the arrangement of Figure 2 has been corrected. This was corrected in the online version on October 9, 2024.
