# Correction: Unassisted single-channel transcolonic endoscopic appendectomy for an appendiceal neuroma

**DOI:** 10.1055/a-2436-2822

**Published:** 2024-10-09

**Authors:** Pei-Rong Xu, Zu-Qiang Liu, Minying Deng, Quan-Lin Li, Pinghong Zhou

**Affiliations:** 1 92323 Endoscopy Center and Endoscopy Research Institute, Zhongshan Hospital Fudan University, Shanghai, China; 2 92323 Pathology, Zhongshan Hospital Fudan University, Shanghai, China



In the above-mentioned article the arrangement of Figure 2 has been corrected. This was corrected in the online version on October 9, 2024.

